# European Association for Palliative Care (EAPC) framework for palliative sedation: an ethical discussion

**DOI:** 10.1186/1472-684X-9-20

**Published:** 2010-09-13

**Authors:** Niklas Juth, Anna Lindblad, Niels Lynöe, Manne Sjöstrand, Gert Helgesson

**Affiliations:** 1 Karolinska institutet, Stockholm Centre for Healthcare Ethics (CHE), LIME, Berzelius väg 3, plan 4, 171 77 Stockholm, Sweden

## Abstract

**Background:**

The aim of this paper is to critically discuss some of the ethically controversial issues regarding continuous deep palliative sedation at the end of life that are addressed in the EAPC recommended framework for the use of sedation in palliative care.

**Discussion:**

We argue that the EAPC framework would have benefited from taking a clearer stand on the ethically controversial issues regarding intolerable suffering and refractory symptoms and regarding the relation between continuous deep palliative sedation at the end of life and euthanasia. It is unclear what constitutes refractory symptoms and what the relationship is between refractory symptoms and intolerable suffering, which in turn makes it difficult to determine what are necessary and sufficient criteria for palliative sedation at the end of life, and why. As regards the difference between palliative sedation at the end of life and so-called slow euthanasia, the rationale behind stressing the difference is insufficiently demonstrated, e.g. due to an overlooked ambiguity in the concept of intention. It is therefore unclear when palliative sedation at the end of life amounts to abuse and why.

**Conclusions:**

The EAPC framework would have benefited from taking a clearer stand on some ethically controversial issues regarding intolerable suffering and refractory symptoms and regarding the relation between continuous deep palliative sedation at the end of life and euthanasia. In this text, we identify and discuss these issues in the hope that an ensuing discussion will clarify the EAPC's standpoint.

## Background

Palliative sedation at the end of life is much debated, since it involves ethically controversial issues regarding the good life and death [1], bordering on hotly contested issues regarding euthanasia and physician assisted suicide. Accordingly, the European Association for Palliative Care (EAPC) Ethics Task Force emphasised the need for a broad and continuous discussion of these questions in 2003 [2]. Recommendations [3,4] and a forum for discussions http://www.eapcnet.org/forum have since been presented.

In 2009 the EAPC authorised a recommendation: EAPC recommended framework for the use of sedation in palliative care (referred to here as the EAPC framework) [4]. The aim of this paper is to critically discuss some of its content. Taken as a whole, we think that the EAPC framework presents excellent procedural guidelines for palliative care. It both motivates the importance of procedural guidelines and pinpoints the key clinical issues needing to be addressed. However, there are a couple of ethically controversial issues where we think that the EAPC framework would have benefited from clarifying its position to a larger extent. In this text we identify and discuss these issues in the hope that an ensuing discussion will clarify the EAPC's standpoint.

Accordingly, we will focus on the parts of the text that are relevant to ethically controversial issues in end of life decisions. More specifically, we will focus on the themes in the text that are relevant to so-called continuous deep sedation, i.e. continuing to alter patient consciousness without specifically intending to discontinue sedation [5], when this is done at the end of life. More specifically still, we think there are two particular questions where the framework would have gained by being more precise: (1) regarding intolerable suffering and refractory symptoms and (2) regarding the relation between continuous deep sedation at the end of life and euthanasia. These issues have been extensively discussed elsewhere (as regards question (1), see e.g. [6,7]; as regards question (2) see e.g. [8,9]), which demonstrates the importance attached to these issues. This in itself constitutes a reason for further discussion about them.

In the following, we will discuss these questions, taking as our point of departure what the EAPC framework has to say about them, and state in what way we think these issues need to be clarified.

## Discussion

### Intolerable suffering and refractory symptoms

Intolerable suffering from refractory symptoms is generally conceived of as an indication for palliative sedation therapy. According to the EAPC framework, in the absence of refractory symptoms, the use of palliative sedation in the end of life is characterised as "abuse": "This [abuse of palliative sedation] may occur by the deliberate use of deep sedation in patients who have no refractory symptoms" (p. 582) [4]. Moreover, the EAPC framework states that "[c]ontinuous deep sedation should be selected first if: ...the suffering is indeed refractory" (p. 586) [4]. In other words, according to the EAPC framework, the presence of refractory symptoms is a necessary condition for an ethically defensible initiation of sedation at the end of life, in particular when there is no intention of discontinuing sedation before the patient dies. Then it becomes crucial to be able to determine which symptoms are refractory.

The EAPC framework characterises refractoriness as "intolerable distress due to physical symptoms, when there is a lack of other methods for palliation within an acceptable time frame and without unacceptable adverse effects" (p. 584) [4]. What should, in turn, be seen as intolerable distress is not made clear. However, in some of the texts on which the framework is based, there are some more precise suggestions. For instance, according to de Graeff and Dean intolerable suffering is "...determined by a patient as a symptom or state that he or she does not wish to endure" (p. 68) [3]. For refractory symptoms the following definition is used (p. 70, our emphasis) [3]:

"A symptom is regarded as being refractory (as opposed to difficult to treat) when the *clinician *perceives that further invasive or non-invasive interventions are (1) incapable of providing adequate relief, (2) associated with excessive and intolerable acute or chronic morbidity, and/or (3) unlikely to provide relief within a tolerable time frame."

According to these descriptions it is the patient who determines when the suffering is intolerable, and the physician (or other clinician) who determines whether a symptom is refractory or not. However, it is not clear why clinicians are in the best position to determine whether or not a symptom is refractory, among other things because it is questionable whether clinicians are in a better position than the patient to determine whether or not an intervention provides "adequate relief". This difficulty has been acknowledged in discussions regarding intolerable suffering. For instance, Thorns writes [10]:

"Suffering and distress are subjective symptoms and so can only really be judged by the patient. It is therefore debatable as to whether the patient or the professional should decide when the symptoms become refractory and whether all palliative treatments need to have been "applied" or just "offered"."

To be sure, the EAPC framework recognises the "subjectivity of refractoriness" (p. 582) [4]. However, the upshot of this is that regarding the vital question as to when palliative sedation therapy is applicable, the EAPC framework leaves the clinicians in the lurch: it is unclear what symptoms are necessary and who the primary party to determine this is: the patient or the clinician.

This problem is accentuated when it comes to so-called psychological or existential suffering. As regards physical or somatic symptoms, the most common refractory symptoms are explicitly identified: "agitated delirium, dyspnoea, pain and convulsions. Emergency situations may include massive haemorrhage, asphyxiation, severe terminal dyspnoea or overwhelming pain crisis" (p. 584) [4]. However, refractory existential and psychological distress as an indication for palliative sedation therapy in the end of life is considered to be so controversial as to merit "special guidelines" (p. 588) [4], including repeated trials of intermittent therapy before continuous therapy can even be considered.

The view that existential distress is a controversial indication for palliative sedation therapy implies that this kind of suffering is regarded as different from somatic symptoms. Even though arguments in favour of treating existential suffering as different from somatic or physical (causes of) suffering are presented (p. 588) [4], it is not self-evident that it should be treated differently. In a text published on the EAPC homepage, regarding decision-making, the concept of suffering is more thoroughly discussed. The authors write [11]:

"Sometimes a distinction is made between physical and existential suffering, yet a case can be made that all suffering is existential. Pain (or any other symptom) and suffering are not inextricably nor inevitably tied to each other, and suffering can exist without physical symptoms."

This can be taken as an argument in favour of not treating somatic and existential suffering differently, or to consider some existential suffering as a refractory symptom itself, at least in the end of life. The EAPC Ethics Task Force seems to hold such a view when defining *terminal *sedation as the use of sedative medication to relieve intolerable suffering during the last days of life (p. 98) [2]. This definition indicates that intolerable suffering, regardless of what kind of suffering we are dealing with, is sufficient for sedation. However, in the article by de Graeff and Dean it is said that "Palliative sedation therapy (PST) is the use of specific sedative medications to relieve intolerable suffering from refractory symptoms by a reduction in patient consciousness" (p. 68) [3]. Obviously, this definition holds refractory symptoms to be something other than mere intolerable suffering, so existential intolerable suffering in the end of life would, accordingly, be an insufficient indication for palliative sedation. Thus, this definition implies that if psychological/existential suffering were to exist unaccompanied by refractory somatic symptoms, then the criteria for PST would not be fulfilled and the treatment not applicable.

As far as we can tell, the question of whether intolerable suffering is a sufficient or only a necessary precondition for initiating palliative sedation is left unsolved in the EAPC framework. The characterisation of refractoriness cited above indicates that it is only a necessary condition, since it states that the intolerable symptoms must be due to physical symptoms. However, the fact that refractory existential and psychological distress is addressed separately as "refractory" (p. 588) [4] seems to indicate that it could be sufficient. What seems to be lacking in the EAPC framework is the very thing you expect from a guideline, namely a more thorough analysis of intolerable suffering, refractory symptoms and the relation between them: what are necessary and sufficient criteria for palliative sedation at the end of life, and why?

### Intending death

In the EAPC framework, reference is made to the discussion regarding so-called "slow euthanasia" (p. 582) [4]. This is described as occurring "when clinicians sedate patients approaching the end of life with the primary goal of hastening the patient's death" (p. 582) [4]. The perceived problem with slow euthanasia does not seem to be the hastening of death in itself, since the EAPC framework is careful to point out the risk of futile treatments or insufficient palliative measures at the end of life as a result of "exaggerated concerns about hastening death" (p. 582) [4]. Moreover, the EAPC readily acknowledges that the proportional use of sedation (i.e. the proportion needed to deal with symptoms and suffering) *could*, in fact, on occasion, hasten death (p. 582) [4]. The problem, rather, occurs when hastening the patient's death is "the primary goal" of sedation. What can be read out of this, we think, is that intending death and using it as a means to relieve intolerable suffering is considered unacceptable by the EAPC framework, since doing so is equated with abuse. This relates to much-debated issues regarding the (1) definition of palliative sedation, (2) the difference between palliative sedation and euthanasia, and (3) the moral relevance of goals or intentions.

If we start with (1) and (2), the definitional difference between palliative sedation in the end of life (including continuous deep sedation) and euthanasia is often cast in terms of intentions, very much as the EAPC framework suggests. For instance, the EAPC Ethics task force on palliative care and euthanasia stated in 2003 that the intention of sedation is to relieve intolerable suffering by using a sedating drug for symptom control, as opposed to euthanasia, where the intention is to kill the patient by administering a lethal drug [2].

According to these definitions, palliative sedation at the end of life is clearly something other than euthanasia. However, the EAPC framework (as well as other texts on this) fails to notice an ambiguity in the concept of intention, the awareness of which makes the initial impression of a vast moral difference between palliative sedation and euthanasia fade somewhat. One way of accounting for the intentions underlying actions is to refer to the ultimate end for which something is done. This is a neat way of accounting for the fact that the intention of palliative sedation is not to shorten or terminate patients' lives: palliative sedation is done *for the sake of relieving symptoms and primarily intolerable suffering of different kinds*. However, the same thing can be said about euthanasia for terminally ill patients: euthanasia is to knowingly kill a person by the administration of drugs, at that person's voluntary and competent request *for the sake of relieving symptoms and primarily intolerable suffering of different kinds*. The ultimate end of euthanasia is not to end the patient's life - i.e., that is not the sake for which the administration of drugs is done. Rather, the ultimate end is to relieve suffering by other means than palliative sedation. Of course, there is still the difference that euthanasia consists of *knowingly *(and in *this *sense intentionally) terminating someone's life, while palliative sedation must not involve anything of the kind. Since this ambiguity of the term intention is not mentioned, the difference between palliative sedation and euthanasia is made to look bigger than it really is. Moreover, this presents a scurrilous portrait of euthanasia by giving the impression that its ultimate aim is to kill off the patient.

It might still be argued that there is something wrong with intentionally (in the sense of knowingly) hastening death that concerns the *means *used to achieve the ultimate end of relief from unbearable suffering. The *principle of double effect *rules out actions with a good and a bad effect where the bad effect is 'directly intended', but permits such actions where the bad effect is not directly intended but merely foreseen. The principle of double effect could arguably be used as an ethical dividing line that approves of palliative sedation but forbids euthanasia, since the latter but not the former directly intends death (as a means to the good end of relieving the patient of unbearable suffering). Accordingly, the principle relates to (3) above: it is a way of trying to justify the moral relevance of intentionality.

However, there are controversies regarding both whether or not palliative sedation in general and continuous deep sedation in particular in the end of life actually fulfils the conditions of the principle of double effect and whether or not it is a plausible principle at all. There are those who argue that the principle can be used as a dividing line between palliative sedation, including deep continuous sedation until death, and euthanasia [10,12]. However, there are those who claim that neither euthanasia nor continuous deep sedation satisfies the conditions of the principle of double effect [13]. These authors argue that continuous deep sedation at the end of life either means the irreversible loss of consciousness or else hastens death, and furthermore seem to presuppose that these effects are always undesirable. However, there are also those who claim, by arguing against these claims, that both euthanasia and palliative sedation are compatible with the principle of double effect. For instance, de Graeff and Dean argue that the principle of double effect is not applicable to palliative sedation, because correctly administered sedation does not shorten life and "because the death of the patient ... is not necessarily untoward" [3].

We are actually sympathetic towards the second of de Graeff's and Dean's arguments: arguably, the principle of double effect is not applicable to palliative sedation when permanent loss of consciousness, which is the result of continuous deep sedation until death, is not 'untoward' for patients. This would be the case when unbearable suffering, as judged by the patient, is an unavoidable consequence of being conscious and this goes for the remainder of her existence. In such a case, it could reasonably be maintained that even the permanent loss of consciousness is not a bad effect of continuous deep sedation till death. That is, unless permanent loss of consciousness is bad for these patients in the situation they are in, continuous deep sedation does not seem to be an unacceptable means of eliminating their unbearable suffering. However, if one accepts this line of reasoning, the allegedly vast moral difference between continuous deep sedation and euthanasia has yet to be explained, since the same line of reasoning can be used to defend euthanasia, at least if there is no morally relevant difference between the permanent loss of consciousness and death (which, we think, has been forcefully argued elsewhere [14]). The same goes, of course, if one rejects the ethical relevance of the principle altogether, as some authors do [15]. Note that nothing of what we have been saying so far once and for all demonstrates that there is no morally relevant difference between continuous deep sedation and euthanasia. We have only claimed that it remains unclear wherein the difference lies and, more importantly, in what way this difference can be accounted for in terms of intentions.

We think that the EAPC framework would have benefited from taking a clearer stand in the discussion regarding slow euthanasia. When, more specifically, does palliative sedation at the end of life constitute abuse? Whenever it hastens death (it would seem not, as we understand it)? If it is intentionally hastening death that is problematic, in what sense of intention? And why is it, then, problematic? That is, according to which principle(s) is it problematic and is this (these) principle(s) reasonable?

## Conclusions

In this text, we have argued that the EAPC framework would have benefited from taking a clearer stand on the ethically controversial issues regarding intolerable suffering and refractory symptoms and regarding the relation between continuous deep palliative sedation at the end of life and euthanasia. It is unclear what constitutes refractory symptoms and what the relationship is between refractory symptoms and intolerable suffering, which in turn makes it difficult to determine what are necessary and sufficient criteria for palliative sedation at the end of life, and why. As regards the difference between palliative sedation at the end of life and so-called slow euthanasia, the rationale behind stressing the difference is insufficiently demonstrated, at least partly due to an overlooked ambiguity in the concept of intention. It is therefore unclear what kind of palliative sedation at the end of life amounts to abuse and why. We hope that an ensuing discussion will clarify EAPC's standpoint regarding these issues.

What we have been saying does not amount to concrete alternative recommendations. However, we hope that we convey a message about the spirit in which we would like to see frameworks on palliative sedation at the end of life written: that the best interest and autonomous decisions of the patients should be the primary concerns. This is not to say that health care professionals have no role in the decision-making regarding palliative sedation at the end of life. They have a crucial role since, for instance, only medical professionals can determine what alternative treatments are available and whether or not the patient is in a terminal stage of illness. Rather, the point is that it must be made clear when and why health care professionals should be allowed to deny intolerably suffering patients at the end of life continuous deep sedation with reference to symptoms not being refractory. In other words, if one cannot say in what sense, when, and why intolerable suffering or refractoriness of symptoms should be determined by someone else than the patient, the patient's judgement on when suffering is intolerable and symptoms are refractory should guide decision-making at the end of life. And if one cannot say in what sense, when, and why the intentions of health care professionals matter, the best interest and autonomous decisions of the patients override considerations about the possible intentions of health care professionals.

## Competing interests

The authors declare that they have no competing interests.

## Authors' contributions

NJ was in principal charge of drafting the manuscript and developing its intellectual content. AL and NL helped with the drafting of the manuscript and its critical revision, particularly the section Intolerable suffering and refractory symptoms. MS and GH helped with the drafting of the manuscript and its critical revision, especially the section Intending death. All authors read and approved the final manuscript.

## Pre-publication history

The pre-publication history for this paper can be accessed here:

http://www.biomedcentral.com/1472-684X/9/20/prepub
